# Neurotropism and behavioral changes associated with Zika infection in the vector *Aedes aegypti*

**DOI:** 10.1038/s41426-018-0069-2

**Published:** 2018-04-25

**Authors:** Julie Gaburro, Asim Bhatti, Jenni Harper, Isabelle Jeanne, Megan Dearnley, Diane Green, Saeid Nahavandi, Prasad N. Paradkar, Jean-Bernard Duchemin

**Affiliations:** 1 CSIRO Health and Biosecurity, Australian Animal Health Laboratory, Geelong, Australia; 2 Deakin University, Institute for Intelligent Systems Research and Innovation (IISRI), Geelong, Australia; 3 School of Medicine, Deakin University, Geelong, Australia

## Abstract

Understanding Zika virus infection dynamics is essential, as its recent emergence revealed possible devastating neuropathologies in humans, thus causing a major threat to public health worldwide. Recent research allowed breakthrough in our understanding of the virus and host pathogenesis; however, little is known on its impact on its main vector, *Aedes aegypti*. Here we show how Zika virus targets *Aedes aegypti*’s neurons and induces changes in its behavior. Results are compared to dengue virus, another flavivirus, which triggers a different pattern of behavioral changes. We used microelectrode array technology to record electrical spiking activity of mosquito primary neurons post infections and discovered that only Zika virus causes an increase in spiking activity of the neuronal network. Confocal microscopy also revealed an increase in synapse connections for Zika virus-infected neuronal networks. Interestingly, the results also showed that mosquito responds to infection by overexpressing glutamate regulatory genes while maintaining virus levels. This neuro-excitation, possibly via glutamate, could contribute to the observed behavioral changes in Zika virus-infected *Aedes aegypti* females. This study reveals the importance of virus-vector interaction in arbovirus neurotropism, in humans and vector. However, it appears that the consequences differ in the two hosts, with neuropathology in human host, while behavioral changes in the mosquito vector that may be advantageous to the virus.

## Introduction

Arbovirus infections transmitted by mosquitoes have great impact on global human health^1^. Among highly diverse arboviruses capable of causing diseases in humans, viruses of the *Flaviviridae* family have the highest prevalence, and hence are of substantial clinical importance to humans^2^. The most widely spread mosquito-borne viruses dengue, West Nile, Japanese encephalitis, and more recently Zika^3^ cause significant morbidity and mortality.

After its discovery in 1947, Zika virus (ZIKV) drew very little attention, being both rare and having mild clinical outcomes^4^. Following the recent outbreaks in the Pacific and the Americas in 2014–2015, studies revealed a strong link between ZIKV infection and neuropathology, i.e., Guillain–Barre syndrome in French Polynesia^5^ and the Americas^6^, congenital syndrome in new born with microcephaly^7^ and ocular abnormalities^8, 9,]^ in South America,^7^ and recent records of encephalitis^10, 11^ and myelitis in adults^12^. Zika virus neurovirulence has been widely studied with different models, in vitro with human pluripotent stem cell (hPSC)-derived neural progenitor cells and organoids^13, 14^, or mouse models^15, 16^ with different viral strains from Africa, Asia and Brazil^8, 17^. As arboviruses, most flaviviruses replicate alternately between mammalian hosts and insect vectors, with no obvious negative consequences for the latter. West Nile virus can infect human neuronal cells, resulting in encephalitis^18^, as well as its mosquito vector’s nervous system^19^. Dengue virus, despite being much less neurotropic in human than WNV, is also neurotropic in mosquitoes^20^, and has been shown to modify mosquito behavior^21^. Although ZIKV shows a strong neurotropism in humans and other mammal models, studies addressing the nervous system infection have so far neglected the vector part of the virus lifecycle. As attested by multiple *Drosophila* models for human neurological diseases^22^, mammal and insect neurons are conserved at many cellular levels^23^. In spite of the obvious differences (placenta, or blood–brain barrier, etc.), viral infection mechanisms could be shared between the two virus hosts and the natural vector host deserves attention to decipher biological processes leading to potential disease mechanisms.

Here we investigate, the behavioral impact of ZIKV infection and its neurotropism in the main mosquito vector *Aedes aegypti* (*Ae*. *Aegypti*). Experiments were performed along with dengue virus serotype-2 (DENV2) as a control. Beside the behavioral assays, we used the microelectrode array (MEA) technology, used for the first time in insect neurons, to record electrical spiking activity from mosquito primary neuron cultures. This technique provides important data about impact of viral infection on neuronal network communication in mosquitoes. Finally, we used confocal microscopy to examine possible network structure modifications and mRNA expression to elucidate possible neurotransmitter cellular pathways involved during ZIKV infection. This study highlights the importance of studying ZIKV infection from the vector’s perspective in order to fully understand ZIKV infection dynamics and host neuronal pathogenesis.

## Results

### Impact of virus infection on vector’s behavior

**Fig. 1:**
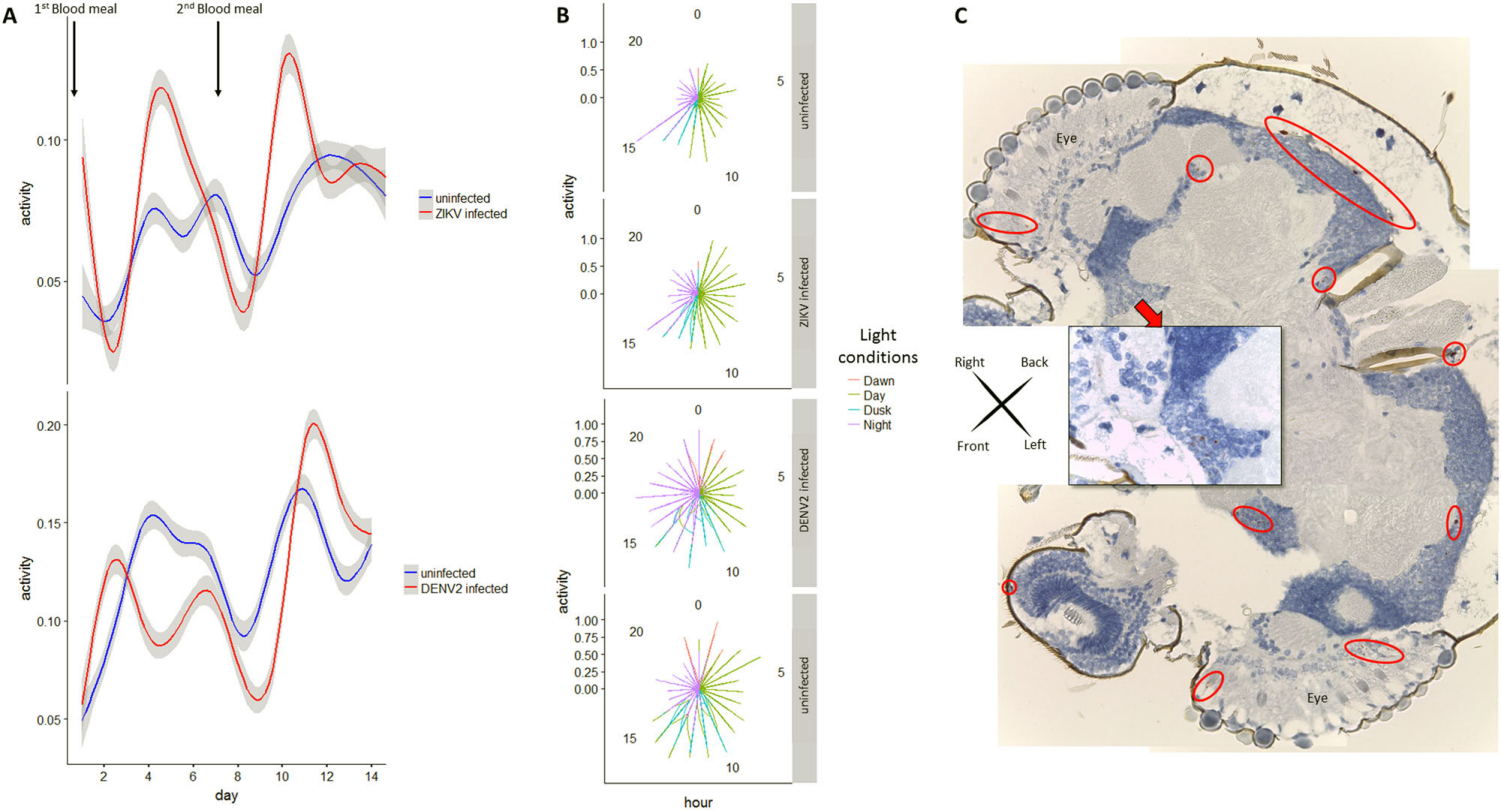
Flaviviruses Zika and Dengue infection effect on *Aedes aegypti*’s behavior and Zika virus neurotropism. **a** Generalized additive modeling for *Aedes aegypti* activity time series analysis (number of movements per frame normalized by the number of females present in the cage) post infectious blood-meal (0 dpi, first blood-meal) and non-infectious second blood meal (7 dpi). Curves are smoothed by generalized additive model based on a cubic regression spline. **b** Graphs with polar coordinates representing females activity in respect to “day” (100% light), “night” (0% light), “dawn” (50% then 10% light) and “dusk (10% then 50% light). **c** Mosaic of head sections of an *Aedes aegypti* female at two weeks post oral-ZIKV infection with pan-flavivirus NS1 detection (red circles for ×4 magnification and red square for ×40 magnification site)

To test for potential impact of virus infection on *A*. *aegypti* mosquito activity, female mosquitoes were recorded by time-lapse video for two weeks after infectious blood meal. Locomotion activity showed 24 h oscillations, being mainly diurnal, with peaks at the beginning and end of each photophase, consistent with previous findings^24, 25^. However, ZIKV-infected females showed significantly increased diurnal locomotion activity compared to uninfected females, especially during the egg-laying, or oviposition phase (Fig. 1a and Supplementary Figure S1C). Dengue virus-infected females also showed an increase in activity, although delayed compared to the ZIKV-infected group. Polar graph illustration also revealed increase in activity for ZIKV-infected females mostly during the day (100% light condition), whereas DENV2-infected females showed increased activity during the night (Fig. 1b). Compared to control, both infected groups showed a wider space occupation in the cage by active females (Supplementary Figure S2). No negative fitness effect of infection was detected either on life expectancy (see survival curves in Supplementary Figure S1B and S3 A and B) or on number of eggs laid (Supplementary Table S1). Altogether, these data indicate a change in behavior of the main vector after natural infection by these two flaviviruses. This indicates possible interplay between virus infection and neural system functioning. Although DENV2 has been detected in the head tissues of *A*. *aegypti* post natural infection^20^, no data are available regarding the neurotropism of ZIKV in its main vector.

### Zika virus neurotropism in mosquito

To confirm the presence of ZIKV in the central nervous systems of orally infected *A*. *aegypti*, sections from female mosquito heads were tested two weeks *post* ZIKV infection using pan-flavivirus anti-non-structural protein 1 (NS1) antibody. Replicating virus, as indicated by positive NS1 staining, was present in 8 out of 11 ZIKV-infected mosquito heads (Fisher exact one-tail test *P* = 0.0256), indicating a tropism of ZIKV to the mosquito central nervous system (brain) as well as peripheral sensory organs (antenna, eyes) (Fig. 1c). This indicates the presence of ZIKV and DENV in mosquito heads possibly disrupting neuronal communication, also attested by the change of behavior observed.

### Virus impact on mosquito neuron spike activity

**Fig. 2:**
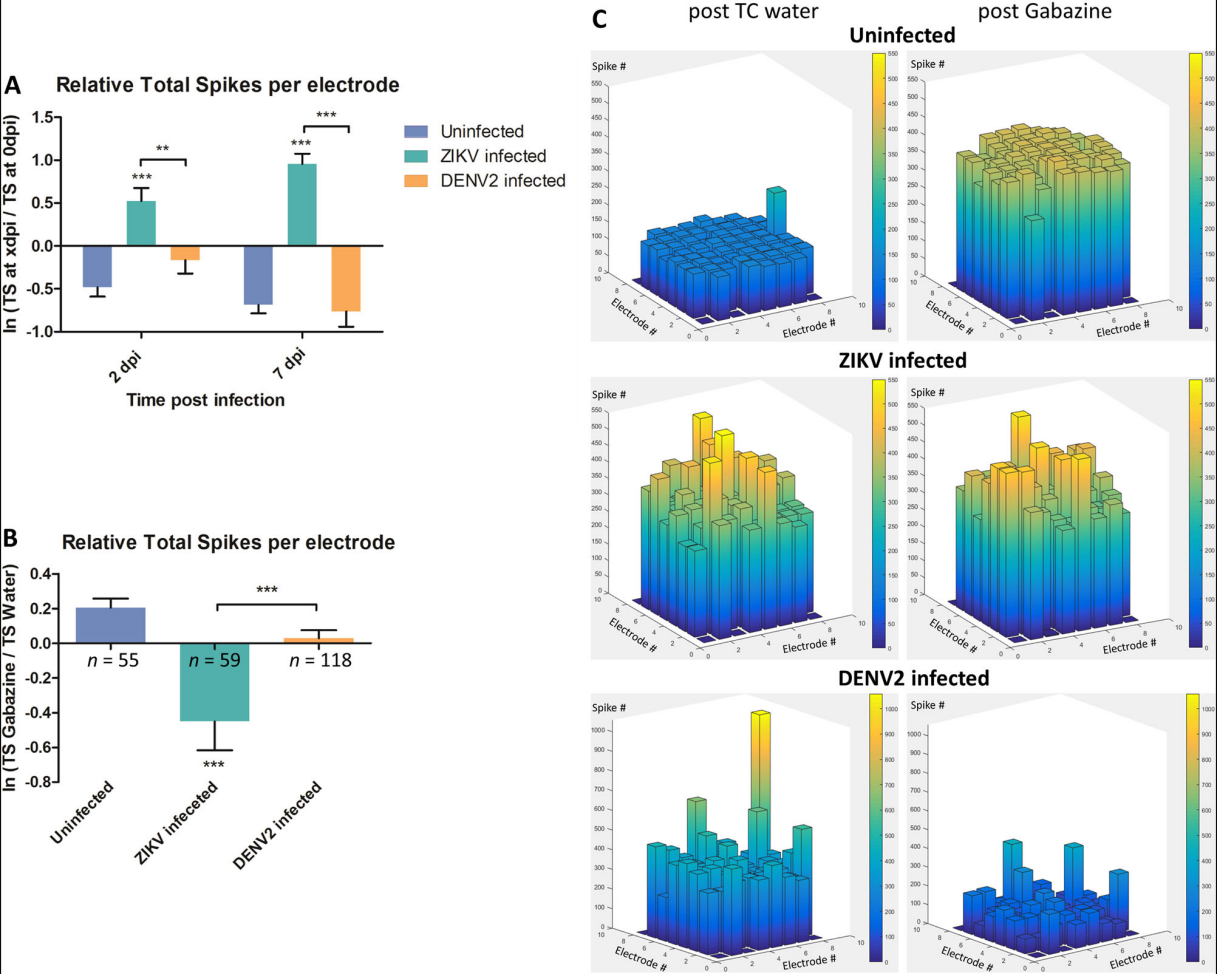
Electrophysiological analysis of infected mosquito primary neuron cultures. **a** Spontaneous spiking activity with average ratio of total spike (TS) number per electrode between 0 dpi (reference) and TS number at 2 and 7 dpi of spontaneous activity. **b** Mosquito neurons stimulated activity with average ratio of TS number per electrode between gabazine stimulus and solvent (water) introduction; *n* = number of active electrodes. **c** 3D electrode activity maps of the effect of gabazine stimulation on mosquito neuronal networks for 2 min, post water as baseline and post gabazine. Statistical differences were calculated with unpaired *t* tests by comparing uninfected group to infected groups unless indicated by bracket. *P < 0.05; **P < 0.01; ***P < 0.001; means ± SEM. For (**a**), *n* values are detailed in Supplementary Table S2

To validate our hypothesis of the ability of flaviviruses to interfere with neuronal communication, electrophysiological activity was recorded at 2, 3 and 7 days post infection (dpi) using planar Microelectrodes Array (MEA)^26^, a method rarely used with invertebrate^27^ or with virus infection. For each individual electrode, the total spike (TS) number was recorded at different times post infection for 30 min. At 2 dpi, ZIKV infection triggered hyperactivity in *A*. *aegypti* primary neuron cultures (Fig. 2a, Supplementary Figure S4 A and B and Supplementary Table S2), which remained hyperactive at 7 dpi. This indicates that ZIKV infection of mosquito neurons leads to sustained hyper excitation. On the contrary, no significant differences were recorded at these time points for DENV2-infected cultures.

GABA_A_ or ɤ-Aminobutyric acid, is an amino acid inhibitory neurotransmitter widely present in the vertebrate and invertebrate nervous system^28, 29^. Cultures were treated with gabazine (Tocris SR 95531 hydrobromide), a GABA_A_ antagonist, to stimulate the firing activity of neuronal networks^30^ and check for the possibility of further spiking excitation. The uninfected neurons showed increased activity after gabazine treatment as shown before^31, 32^ (Fig. 2b). Cultures infected with ZIKV had significantly lower response to gabazine compared the solvent (Fig. 2b and Supplementary Figure S5 A and B), due to the high spontaneous spiking activity triggered by the infection. This result is also confirmed by the network activity illustrated by the 3D electrodes maps (Fig. 2c), where the spike number per electrode is comparable post solvent and gabazine in ZIKV-infected cultures. Indeed, Zika virus-infected mosquito cultures showed hyper reactivity of neurons in response to water, as well as to gabazine.

### Virus dynamics and impact on mosquito primary neuronal network

**Fig. 3:**
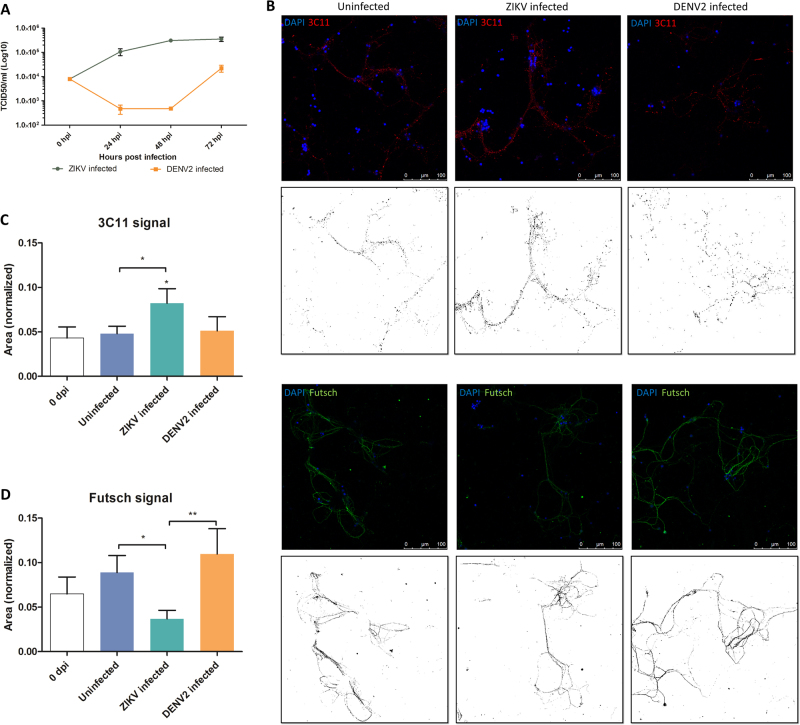
Effect on neuronal network after Zika virus (ZIKV) and Dengue serotype-2 virus (DENV2) infection in primary neuron cultures. **a** ZIKV replication dynamics (using TCID_50_ in Vero cells) in primary neurons from mosquito primary neuron cultures (*n* = 3 per condition). **b** Confocal images of mosquito primary neuron culture *post* ZIKV and DENV2 infection at 7 dpi. In red is 3C11 antibody, corresponds to Synapsin-1, and in green is Futsch antibody, a MAP1B homolog. Each confocal image has its respective image showing stained signal after applying image threshold with ImageJ. Quantification of 3C11 signal (**c**) and Futsch puncta (**d**) in mosquito neuron cultures, normalized by the number of DAPI counts. Statistical differences were calculated with Mann–Whitney U tests by comparing 0 dpi to the other groups unless indicated by bracket. *P < 0.05; **P < 0.01; ***P < 0.001; means ± SEM. For (**c**) and (**d**), *n* values are detailed in Supplementary Table S4

TCID_50_ was performed using culture supernatant to confirm if there was virus replication in mosquito primary neuron culture model, as seen in in vivo-infected mosquito heads. Results showed that ZIKV replicated in mosquito primary neuronal cultures by quickly reaching a plateau within 2 dpi (Fig. 3a), whereas DENV2-infected cultures showed an initial decrease followed by increase in virus titers until 3 dpi.

**Fig. 4:**
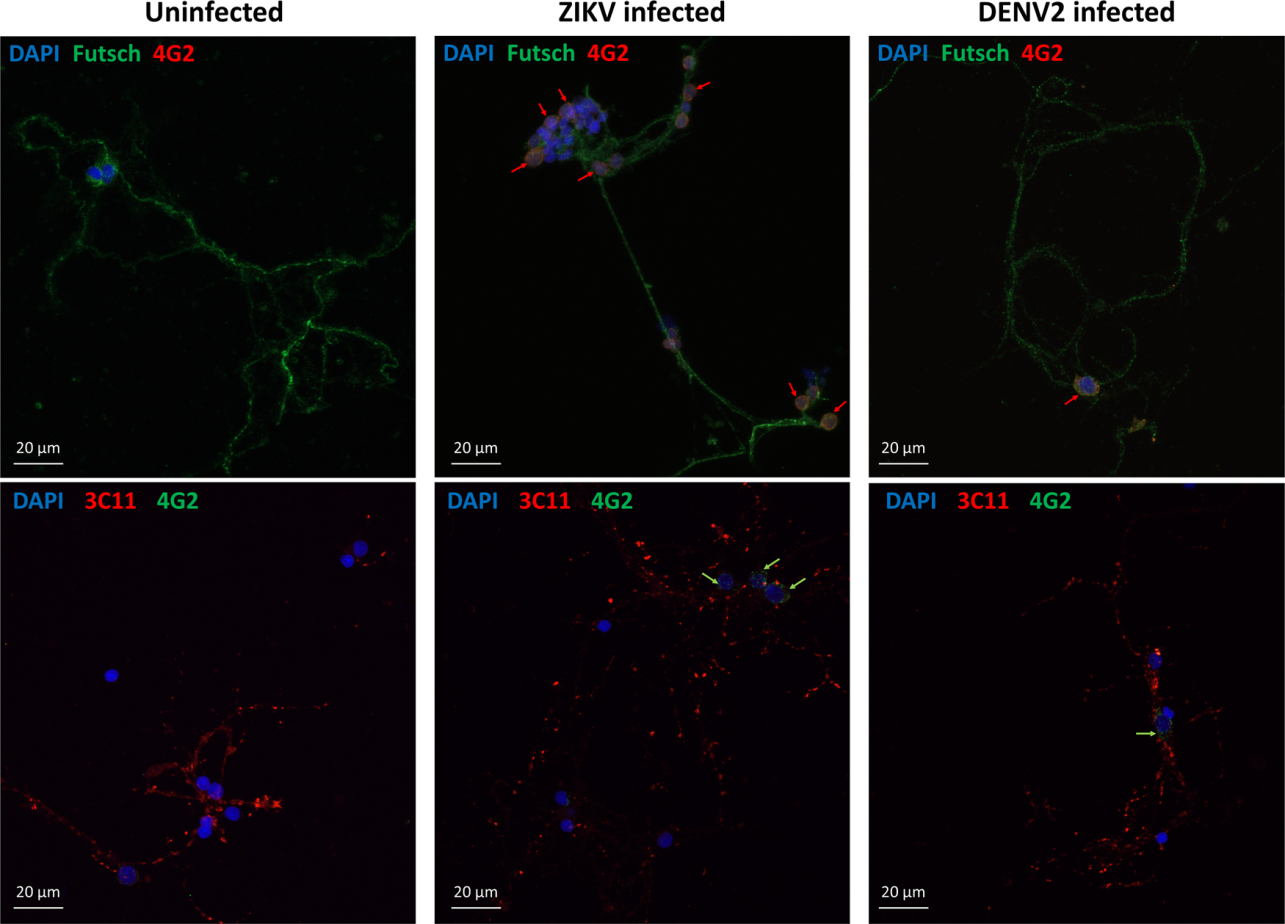
Zika and dengue viruses are present in *Aedes aegypti* primary neuron cultures at 7 dpi. Confocal images of mosquito primary neurons infected with ZIKV or DENV2. Virus signal is indicated by arrows next to the cell nuclei (DAPI in blue) either in co-staining with Futsch or Synapsin proteins (3C11)

Confocal microscopy was performed to compare the effect of the two flaviviruses on the neuronal network’s structure. Synapsin proteins have several functions at the synapse level, including regulation and maintenance of reserve pool of pre-synaptic vesicles for neurotransmitter release^33^. At 7 dpi, ZIKV-infected mosquito cultures showed significantly higher signal of Synapsin compared to the uninfected (Fig. 3b,c). At the same time, there was no difference in DENV2-infected cells compared to control. On the contrary, the antibody signal for Futsch at 7 dpi, a key effector in *Drosophila* synapse development^34^, orthologous to the vertebrate Microtubule Associated Protein-1B (MAP1B)^35^, increased in uninfected and DENV2-infected mosquito cells (Fig. 3b,d), but not in ZIKV-infected cultures. In addition, the presence of the viruses in the primary neuron cultures was confirmed at 7 dpi with a pan-flavivirus envelop antibody (Fig. 4), indicating infection of cells and showing that ZIKV and DENV2 are localized around nuclei of the infected neurons.

### Neurotransmitter pathways response after ZIKV infection

**Fig. 5:**
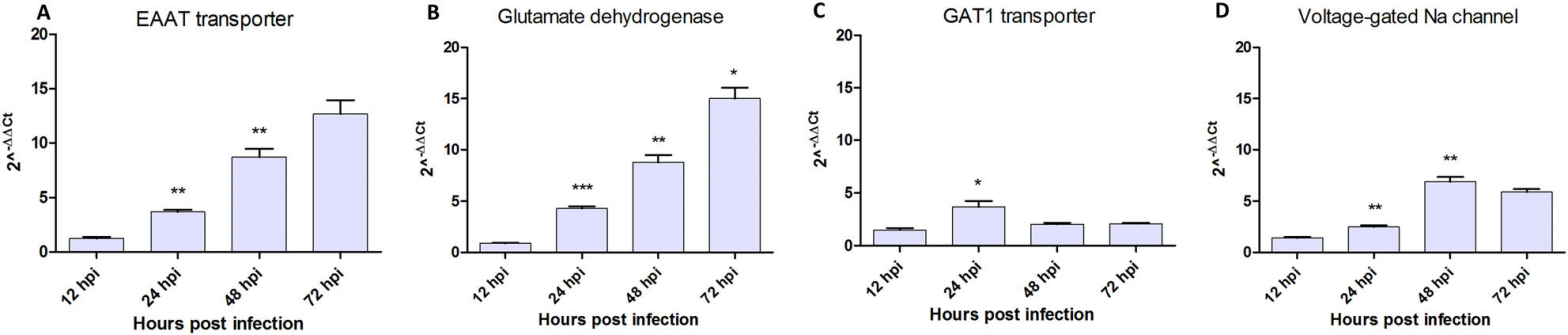
Genes mRNA expression after Zika virus infection in *Aedes aegypti* primary neuron cultures. Quantification of (**a**) Glutamate transporter (EEAT), (**b**) Glutamate dehydrogenase (GD1), (**c**) GABA_A_ transporter (GAT1) and (**d**) Voltage-gated sodium channel (VGNaC) mRNA expression. Statistical differences were calculated with unpaired *t* tests by comparing mRNA expression at time_*i*_ to time_*i*−1_ within each group (*n* = 3). *P < 0.05; **P < 0.01; ***P < 0.001; means ± SEM

Since glutamate is the main excitatory neurotransmitter^36^, it could be responsible for the hyper excitatory effect initiating at 2 days post ZIKV infection. Real-time RT-qPCR performed on ZIKV-infected neurons showed that mRNAs expression of glutamate pathway genes such as the Excitatory Amino Acid Transporter (EAAT) at post-synaptic level and Glutamate Dehydrogenase (GD1) a mitochondrial enzyme responsible for glutamate oxidation and recycling, increased during the first three dpi (Fig. 5a,b). On the other hand, VGlut (Vesicular Glutamate Transporter, a cytoplasmic pre-synaptic glutamate transporter) mRNA showed no substantial change over time after infection (Supplementary Figure S6A). Regarding the GABA_A_ pathway, pre-synaptic GABA_A_ transporter 1 (GAT1) mRNA was significantly over-expressed only transiently in ZIKV-infected neurons at 24 hpi (Fig. 5c) and the GABA_A_ receptor subunit expression was decreased in the same time window (Supplementary Figure S6B). Finally, VGNaC (Voltage-gated sodium channel, responsible for the propagation of action potential) mRNA expression increased in mosquito neurons with a peak at 48 hpi or 2 dpi (Fig. 5d), corresponding to the hyper-excitatory phase recorded with MEA (Fig. 2a). These results indicate that ZIKV infection leads to differential gene expression of glutamate pathway genes in neurons.

## Discussion

Our results reveal behavioral modifications caused by Zika infection in its main vector *A*. *aegypti*, by increasing its locomotor activity as well as increasing its use of the cage space. These behavioral changes post infection underlie a potential way to manipulate the mosquito host and to increase viral transmission, possibly by improving space exploration of infected females. This could be advantageous for the virus, broadening the spatial range of infected vectors and increasing the chances of transmission to new susceptible hosts. It also complements the ZIKV vertical transmission ability from infected mosquito female to its progeny^37, 38^ in a way of increasing the range and numbers of mosquito carrying the virus and able to transmit it to a new mammal host. Changes in mosquito locomotion activity have been found with DENV2 infection in *A*. *Aegypti*^21^ though with different methods (infection bypassing the midgut by intrathoracic inoculation and small volume cage by Drosophila Activity Monitor TriKinetics^®^ techniques). There are only few reports of virus changing its vector behavior^21, 39, 40^. West Nile virus, a neurotropic flavivirus in humans and horses, does decrease the host-seeking behavior of its vector *Culex quinquesfasciatus*, hence with a potential negative effect on virus transmission. Changes of behavior have been also observed in insect-borne plant viruses, which can influence their vectors to enhance their transmission, either indirectly, through the infected host plant, or directly, after acquisition of the pathogen by the vector^41^. These manipulation strategies to enhance virus or pathogen transmission is thus not an uncommon phenomenon. A recent study observed behavioral changes in congenitally ZIKV-infected mice, which showed motor incoordination and visual dysfunctions^42^. These behavioral deficits, detected in mammalian hosts add another proof of the ability of ZIKV to interfere into the CNS.

Interestingly, though both viruses have been detected in neuron cultures (Fig. 4), a strong difference in DENV2 and ZIKV virus infection was observed at the neuronal network level on our mosquito primary cultures. First, ZIKV-infected primary neurons showed an increased spiking activity from 2 to 7 dpi, while DENV2-infected cultures were not different from uninfected ones in electrical activity. Secondly, no significant network structural changes were detected in DENV2-infected culture, while ZIKV-infected neurons showed significantly more synapse connections, but less microtubule protein signals. Since glutamate is the main excitatory neurotransmitter in vertebrates^36^, it is a good candidate to explain this increase of neuronal activity. However, glutamate can also be neurotoxic at high concentrations, triggering trans-neuronal degeneration with damage or death of neighboring neurons connected by glutamatergic synapses^36^. This hypothesis has been proposed, pro parte, for Zika pathogenesis in a mouse embryo model^43^ as well as in alphavirus encephalomyelitis^44^. Our results showed upregulation of EEAT in ZIKV-infected mosquito neurons, which, in cerebral ischemia in humans, has shown to be protective^45^. In human cases of West Nile virus-induced acute flaccid paralysis, and in related hamster model, the EEAT expression is decreased in the spinal grey matter and associated with pathology^46^. In *A*. *aegypti* neuron cultures, the glutamate transporter response could compensate for glutamate overproduction. Similarly, mitochondrial GD1 upregulation would be beneficial to mosquito neurons by increasing catabolism of glutamate towards the tricarboxylic acid cycle^47^. These mechanisms, among others, could allow the mosquito neurons to be tolerant to ZIKV-induced hyper excitation, contrary to mouse neurons^43, 48^^, unpublished^.

The voltage-gated sodium channel (VGNaC or Nav) was found to be upregulated during ZIKV infection of mosquito primary neuron cultures, potentially triggering or sustaining an excitatory state of the mosquito. Interestingly, if insecticide resistant *A*. *aegypti* mosquito carry double *kdr* mutations of the voltage-gated sodium channel (VGNaC or Na_v_), its locomotion activity is increased^49^. Moreover, this channel has been proposed as target of human anti-ganglioside antibodies in Guillain–Barré syndrome associated with *Campylobacter jejuni* infection^50, 51^ by molecular mimicry.

Here, our results show relatively benign and possibly beneficial interaction between ZIKV and mosquito vector, in strong contrast with its deleterious impact on human embryos. These results suggest the idea of ZIKV neurotropism being selected in its vector, with pathological impact on its vertebrate host. Our study underlines the importance of such vector-directed approach in dual hosts systems like arboviruses and opens up new paths for understanding human arboviral pathogenesis.

## Materials and Methods

### Viruses preparation and strain

We used Zika virus (ZIKV) human isolate from Cambodia 2010 (Genbank KU955593) passaged in C6-36 cell line and harvested at 72 h post infection. Dengue virus serotype 2 ET300 (DENV2) isolated from a soldier in Australia returning from East Timor, was passaged 7 times in C6-36 cell line.

### Mosquito rearing and oral infection

Originating from Brisbane, Australia in 2015, *Aedes aegypti* (*A*. *Aegypti*) mosquito colonies are kept in a PC3 laboratory. Colony room temperature was set at 26 °C and 60–70% humidity, with a 14–10 h light–dark photoperiod with gradual two hours of dim light transition mimicking dawn and dusk to avoid startle response induction. Mosquitoes were regularly blood fed (Hemotek membrane feeding system®) with chicken blood. At one week post emergence, young females were given an infectious blood meal for one hour at 37 °C.

Prior blood feeding, plasma was removed and substituted with same volume of virus solution in L15 media to a final 10^4.9^ TCID_50_/ml for ZIKV and 10^5.9^ TCID_50_/ml for DENV2 of viral titer. In control blood meal, L15 media without virus was used. After feeding, only engorged females were kept and sacrificed at two weeks post infection (pi), for more details see^52^.

### Mosquito activity monitoring

Fifteen blood-fed *A*. *aegypti* females, for each uninfected and infected, were isolated in individual Plexiglas cages (30 × 30 × 30 cm) and monitored by a camera with infrared capture capacity (Flea3, Point Grey Research, Canada) as shown in the setup (Supplementary Figure S1A). Recording started at one day post infection (dpi), capturing one frame every 60 s for 14 days. Recording was paused for 3 h at 7 dpi, allowing the intake of a second, not infected, blood meal. Each cage contained a plastic see-through container with a strip of sand paper allowing the females to lay their eggs. Each day had the following light pattern: 0% of light as “night” (for 8 h), 10% of light (for 1 h) then 50% of light (for 1 h) as “dawn”, 100% of light as “day” (for 12 h) and 50% of light (for 1 h) then 10% of light (for 1 h) as “dusk”. An indirect Infrared light facilitated “Night” time recording. Pictures were then converted into a time-lapse video, which was processed by a custom-developed software to monitor mosquitos’ position variations across video frames. The data were then normalized at each time point with the number of females alive in the cage (see survival curves Supplementary Figure S1B). The experimenter manually detected the time instances of ovipositions, defined as presence of females in the container, by watching the recorded videos. The data were exported and analyzed with R statistical software^53^. The algorithm also gives the two dimensions (2D) location of the females when they move during the recording to visualize space occupation linked to their activity. Finally, the eggs were collected after the end of each oviposition period and counted with Icount tool^54^.

### Immunohistochemistry (IHC)

Eleven ZIKV-infected females and four controls were tested for ZIKV presence in head section via IHC using antibody against the Murray Valley Encephalitis Virus NS1 protein (4G4, Roy Hall, University of Queensland) at a dilution of 1:100. Following application of the primary antibody, EnVision FLEX+ Mouse Linker (Dako, Glostrup) was applied to amplify the signal. A HRP labeled secondary antibody, EnVision FLEX/ HRP (Dako, Glostrup), was then applied with the AEC chromagen, AEC+ High Sensitivity Substrate Chromogen (Dako, Glostrup), as a brownish-red precipitate. Prior to immunohistochemical staining, sections were antigen retrieved using the Dako PT link and EnVision FLEX Target Retrieval Solution High pH (Dako, Glostrup) for 20 min at 97 °C. A 3% hydrogen peroxide (VWR) solution in EnVision FLEX Peroxidase- Blocking reagent (Dako, Glostrup) was also used to eliminate endogenous peroxidase activity within the mosquitoes. Slides were examined using a Leica DM500 inverted microscope, and photographed using a Leica digital camera and LAS 4.9.0 software (Leica Microsystems, Germany), magnifications ×4 and ×40.

### Primary neurons dissociation

Adult female mosquitoes, *A*. *aegypti*, were cold killed at 3–4 days post emergence and brains were dissected then isolated in L15 media supplemented with 10% Tryptose Phosphate Broth, 10% Fetal Calf Serum, fungizone and penicillin-streptomycin (50 units/ml). Fifteen to twenty brains were pooled and transferred into a 2 ml tube with clean media for mechanical dissociation by repetitive trituration. Cells were then centrifuged 2 times (5 × *g* for 3 min) and then resuspended in 50 µl of clean media.

### Primary neuron cultures and infection

Primary neuron cultures for confocal microscopy assays, TCID_50_ and RT-PCR were prepared as previously described. For confocal microscopy, coverslips were pre-coated with 100 µl of polyethyleneimine (PEI, Sigma, 0.05% in Borate-buffered solution) at room temperature (RT) for 30 min, rinsed three times with tissue culture treated water (TC-water) and let to dry out in the Biosafety Cabinet class II (BSCII). Cells were plated in a 24-well plate with 25 µl from a stock solution of 3 × 10^6^ cells/ml. Plates were placed in incubator at 28 ºC for half an hour for cells to adhere before adding 1 ml of completed L15 media (10% FCS).

For primary neuron cultures on microelectrode array (MEA), the method was modified from Hales et al.’s^55^. Microelectrode array were pre-coated before cell seeding described before. The coating consists of 100 µl PEI at RT for 30 min, followed by TC-water rinses (3 times); laminin (0.02 mg/ml, Sigma L-2020) was finally added for 20 min at 37 ºC and 5% CO_2_, just before plating. Cells were plated in the center of the MEAs at 7.5 × 10^4^ cells per device (25 µl volume). After half an hour in the 28 ºC incubator for cells to adhere to the bottom of the MEA well, 1 ml of cell media was added. Half of the culture medium was changed every 3–4 days excluding the day before recording.

All primary neuron cultures were infected with the Multiplicity of Infection (MOI) of 0.2 at 7 days of *in vitro* (div) culture, after network maturation. For infections of 24-well plate cultures, the whole media was removed and replaced with 1 ml of virus. For infection on MEAs, cells were infected with 0.2 MOI virus in 300 µl for one hour before adding fresh media.

### Viral titration with TCID_50_

For virus dynamics titration, 1 ml of supernatant from neuron cultures was sampled in triplicates at 24, 48 and 72 h *post* infection (hpi) and stored at −80 ºC till TCID_50_ assays.

Viral titers at the different time points were determined by end point titrations (TCID_50_) in Vero monolayer cell cultures. Each plate was screened for cytopathogenic effect at 3 and 5 days post infection and to determine TCID_50_ using Spearmann–Karber calculation method^56^.

### Quantitative real-time RT-PCR (Q-PCR)

Total RNA was collected from cells at 12, 24, 48 and 72 h post infection (hpi), for gene expression quantification. For this, media was removed and replaced with 200 µl fresh media for each cell type. Cells were removed from the bottom by gentle scraping and vigorous pipetting. Samples are kept at −80 ºC till RT-PCR assays.

Total RNA was extracted using RNeasy Plus Mini Kit (Qiagen Sciences, Maryland, MA) and 10 µl of RNA was used to prepare cDNA using random hexamers and Superscript-III reverse transcriptase (Thermo Fisher Scientific Inc. Australia) as per manufacturer’s protocol. Real-time PCR assay in triplicates was performed using the SYBR® Premix Ex Taq™ II (Takara-Bio Inc, China) and running on a QuantStudioTM 6 Flex real-time PCR System (Applied Biosystems) suing specific primers (see Supplementary Table S3). Settings were: 95 °C for 30 s, followed by 45 cycles of 95 °C for 5 s, 55 °C for 40 seconds, and finally followed by melt-curve stage for fluorescence measurement.

The 2^ΔΔCt^ values were calculated at each time point for each gene as the fold increase over uninfected control at the same time point.

### Immunofluorescence (IF)

At 0 and 7 days post infection (dpi), primary neuron cultures grown on coverslips were fixed using 4% paraformaldehyde in 0.05 M Phosphate Buffered Saline (PBSA) for 20 min with gentle shaking. The coverslips were washed gently three times for 5 min using 1 ml of PBSA. Cells were permeabilized with 1 ml of 0.1% Triton X-100 (Sigma-Aldrich) in PBSA for 10 min and rinsed three times in PBSA. Non-specific binding was blocked with 0.5% BSA in PBSA for 30 min. Primary antibodies were diluted in 0.5% BSA in PBSA and incubated for 1 h at room temperature. The following primary antibodies were used: DAPI (32670, Sigma-Aldrich), 1:50 mouse anti Futsch (DSHB University of Iowa), 1:100 mouse anti 3C11 (DSHB University of Iowa), 1:200 human 4G2 anti pan-flavivirus (Ab00230-10.0, Focus Bioscience). Coverslips were washed three times with PBSA for 5 min. Coverslips were then incubated in species-specific secondary antibodies diluted in 5% BSA in PBSA for 1 h at room temperature, followed by two washes of 5 min with PBSA. Coverslips were then washed twice with water and counterstain nuclei with DAPI for 10 min, before a final wash in water and mounted carefully on glass slides. The slides were observed with a ×63 objective (with oil) using a Leica SP5 confocal microscope for Futsch and 3C11 signal quantification and a ZEISS LSM 800 confocal microscope for virus detection in the cultures. For the number of coverslips and images taken per treatment and condition to quantify the Futsch and 3C11 antibody signals, see Supplementary Table S4.

To quantify Futsch and 3C11 signals, raw images were extracted with LAS AF Lite software (Leica Microsystems, Germany) in single channel format for analysis. Image analysis is done with ImageJ software^57^. Images were first converted into 16-bits format with default threshold (dark background, B&W parameters). Results were normalized with the number of cells by count of DAPI particles. For DAPI counting, the option “Watershed” was applied before analysis to avoid counting merged particles. The Plugin “Analyze particles” was then used with the parameters “0.00–1.00” for circularity and “50-Inf” for size. Futsch and 3C11 signal quantification analysis were done with the automatically calculated value of the “Measure’, and “Raw integrated density” options. The presence of the viruses in the primary neuron cultures was assessed by using the ZEN software from Zeiss microscopy, with the Z-stack capture option. An optical section area was captured every 0.55 µm on a total section distance of 6 µm. The final image was generated with the maximum intensity projection from the Extended Focus module to obtain a depth of field from the previously acquired Z-stacks.

### Data acquisition with microelectrode array

Recording material: microelectrode array (MEA) were used for electrophysiological recording of neuronal networks (60MEA 200/30iR-Ti-gr, Multi-Channel Systems, Reutlingen, Germany). The glass devices consist of 59 TiN/SiN planar round electrodes (10-30 µm diameter; 100-200 µm center-to-center inter-electrode intervals) arranged in a square grid without corners. A single larger electrode served as reference ground electrode, replacing one recording electrode. Online cellular activity was recorded using the MEA60inv System (Multi Channel Systems, Reutlingen, Germany). Action potential was recorded at each electrode sampled at 10 kHz. MC_Rack software (Multi Channel Systems), installed on the acquisition computer, allowed files acquisition. Data analysis was performed off-line using MC_Rack software by Multi Channel Systems and NeuroSigX software developed by researchers at Institute for Intelligent Systems Research and Innovation, Deakin University, Australia.

Recording method: all manipulations and recordings were made in a Biosafety Cabinet class II (BSCII), for safe containment of viruses. Each recording session consisted of a 30 min of spontaneous activity recording at 0, 2, 3, and 7 dpi or 15 min of each post stimulus activity recording at 8 dpi for gabazine stimulation assays. Every record started 5-10 min after placing the MEA on the amplifier to avoid neuron response to mechanical stress due to movement and to allow adaptation to the BSCII conditions and temperature set by the adaptor instead of incubator. MEA’s temperature was maintained at 28 ºC through the recording according to the neuronal culture’s viable temperature. Half of the media was changed after each recording session.

The raw continuous voltage traces were filtered to remove the traces of field potential below 200 Hz generated by the collectively charged network. The high pass filter was comprised of a 2nd order Butterworth filter with cutoff frequency of 200 Hz. The spike detection threshold was set at 8 times the high-pass filtered signal’s standard deviation^58^ (as measured by MC_Rack software, 22 µV) within a 500 ms window.

Recording time points: once the dissociated neurons from mosquitoes were plated on the MEA, the cultures were let to grow and maturate for seven days. At 7 days *in vitro* (div), *Ae. aegypti* primary neuron networks were infected with ZIKV or DENV2 (used as a control) at a MOI of 0.2.

For each experimental assay, six electrophysiological recordings of spontaneous activity were made. The first was done at 7 div before infection and set as reference for further analysis. After infection, records were sampled a 2, 3 and 7 days post infection (dpi). The last recording took place at 8 dpi for stimulation with 20 µM of gabazine^30^, a GABA_A_ antagonist (Tocris SR 95531 hydrobromide [2-(3′-carboxy-2′-propyl)-3-amino-6-p-methoxyphenylpyridazinium bromide]). Before the introduction of the gabazine, a prerecording was done by adding the same volume of solvent (TC-water) in the well as a reference for the analysis of the gabazine stimulus.

### Microelectrode array data analysis

Microelectrode array data analysis with MC_Rack: MEA data analysis is performed offline with MC_Rack software (Multi Channel Systems, Reutlingen, Germany). Active electrodes (AE) are selected if the spike rate for the electrode is equal or higher than 0.01 spike per second. Burst electrodes are detected with the following parameters: maximum interval to start burst = 100 ms, maximum interval to end burst = 100 ms, minimum interval between bursts = 100 ms, minimum duration of burst = 10 ms, and minimum number of spikes in burst = 3 (adapted from^58^, see Supplementary Figure S7). The relative total spike (TS) number changes per electrode is calculated by: Ratio = ln (TS at time point dpi)/(TS at 0 dpi) for spontaneous spike activity. For post gabazine analysis, the TS reference is changed with the activity post stimulation by solvent (TC-water).

Microelectrode array data analysis with NeuroSigX: MC_Rack files are cut into 6 filtered files of 5 min for spontaneous activity at the different times post infection and 6 files of 2 min post gabazine stimulation at 8 dpi. Mcd files are then converted into txt files (MC_Data tool, MultiChannel System, Reutlingen, Germany) and analyzed by NeuroSigX. The NeuroSigX software uses novel spike sorting and data analysis algorithms to explore the neural spike activity and spatio-temporal behavior of the neuronal network^59^. A threshold of 22 µV is employed to maintain the analytical consistency between the preliminary analysis by MC_Rack software and analysis by NeuroSigX. Electrode activity maps figures are extracted for spatial analysis and illustration.

### Statistical analysis and graphics

Graphs and statistical analysis are done using GraphPad Prism 5 software. All statistical tests are done using a two-tailed analysis except when one-tailed mentioned. All statistical results are expressed with the *p* value using the following annotations: ns for *P* > 0.05, *P ≤ 0.05, **P ≤ 0.01, and ***P ≤ 0.001. For mosquito’s behavioral activity analysis, modeling and statistical analysis were done using the R software version 3.4.0^53^ with “ggplot2”^60^ and “mgcv”^61^ packages to perform generalized additive modeling for a mosquito activity time series analysis. The principle behind GAM is similar a regression one, a method which models the response variable by independent variable(s), except that instead of summing effects of individual predictors, GAM calculates a sum of smooth functions. These functions allow to model more complex patterns, and they can be averaged to obtain smoothed curves that are more generalizable. Default parameters were used; however, the robustness of models was checked by stretching the parameters causing no significant change of the results. Finally, polar analysis was done using the “forecast”, “sugrrants” and “tidyverse” packages, with female activity data classified in 4 day periods depending of light conditions (day, night, dawn and dusk).

### Data availability

The data that support the findings of this study are available from the corresponding author upon reasonable request.

## Electronic supplementary material

Supplementary Figure S1

Supplementary Figure S2

Supplementary Figure S3

Supplementary Figure S4

Supplementary Figure S5

Supplementary Figure S6

Supplementary Figure S7

Supplementary Table S1

Supplementary Table S2

Supplementary Table S3

Supplementary Table S4

Read-me
