# Supplementary material for: Neurotropism and behavioral changes associated with Zika infection in the vector Aedes aegypti
Source: Emerg Microbes Infect. 2018 Apr 25;7:68. doi: 10.1038/s41426-018-0069-2 (PMC5915379; doi:10.1038/s41426-018-0069-2)
Supplement: Supplementary file 1 — Supplementary Figure S1 [file 41426_2018_69_MOESM1_ESM.pdf]

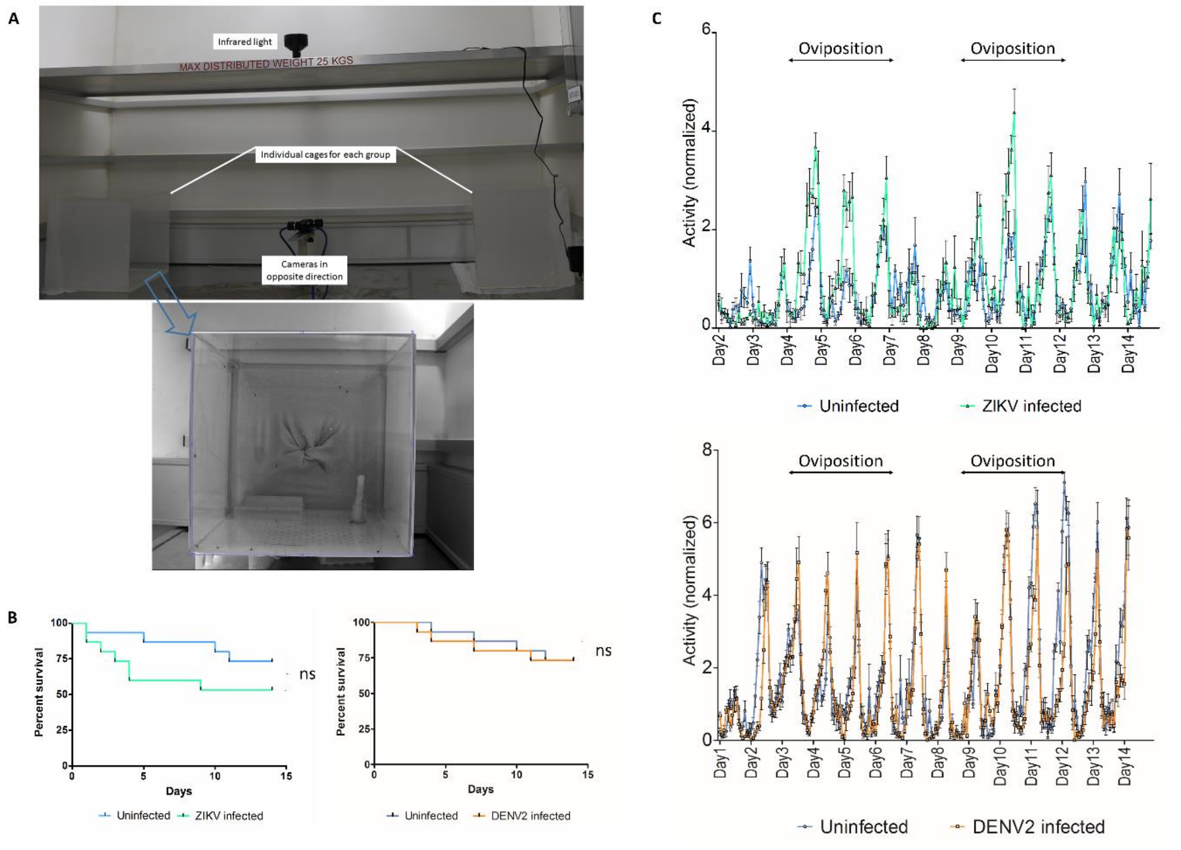

Supplementary Figure S1. *Aedes aegypti* activity monitoring post infectious blood-feeding. (**A**) Experimental set up with the two groups (uninfected and infected females) being recorded in parallel and the camera's view point before launching custom developed algorithm. (**B**) Survival curves of female groups with no significant differences between uninfected and ZIKV or DENV2 infected females (Gehan-Breslow-Wilcoxon Tests). (**C**) Raw data line charts of uninfected and ZIKV or DENV2 infected mosquito females during the day (100% light) showing 24 hours oscillations. Each data point represent one hour average of the sum of 10 minutes recording (±SEM). Two ZIKV and DENV2 infected groups are significantly different than uninfected with respectively P = 0.0142 (Two-tailed Wilcoxon signed rank test, W = -2668) and P < 0.0001 (Two-tailed Wilcoxon signed rank test, W = 7342).
