# Supplementary material for: Neurotropism and behavioral changes associated with Zika infection in the vector Aedes aegypti
Source: Emerg Microbes Infect. 2018 Apr 25;7:68. doi: 10.1038/s41426-018-0069-2 (PMC5915379; doi:10.1038/s41426-018-0069-2)
Supplement: Supplementary file 2 — Supplementary Figure S2 [file 41426_2018_69_MOESM2_ESM.pdf]

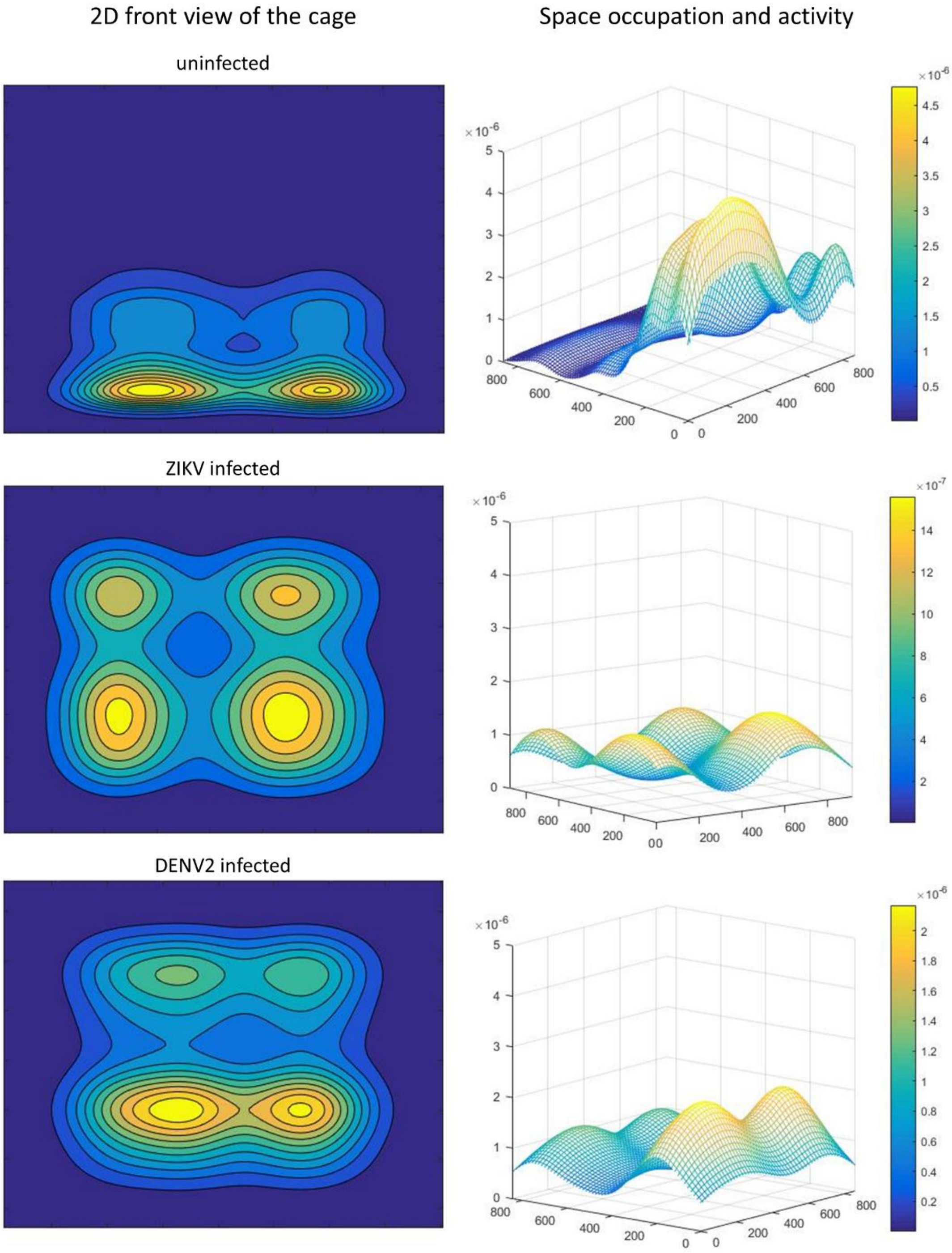

Supplementary Figure S2. ***Aedes aegypti*** **cage space exploration at 5 days post infection.** The 2 dimensions (2D) view of the cage images show where females have been the more active (in yellow) to inactive or absent (in blue) during the time-lapse video recorded at 5 dpi. The corresponding graphs on the right shows the respective space occupation but with the female’s activity added on the y-axis.
