# Supplementary material for: Neurotropism and behavioral changes associated with Zika infection in the vector Aedes aegypti
Source: Emerg Microbes Infect. 2018 Apr 25;7:68. doi: 10.1038/s41426-018-0069-2 (PMC5915379; doi:10.1038/s41426-018-0069-2)
Supplement: Supplementary file 3 — Supplementary Figure S3 [file 41426_2018_69_MOESM3_ESM.pdf]

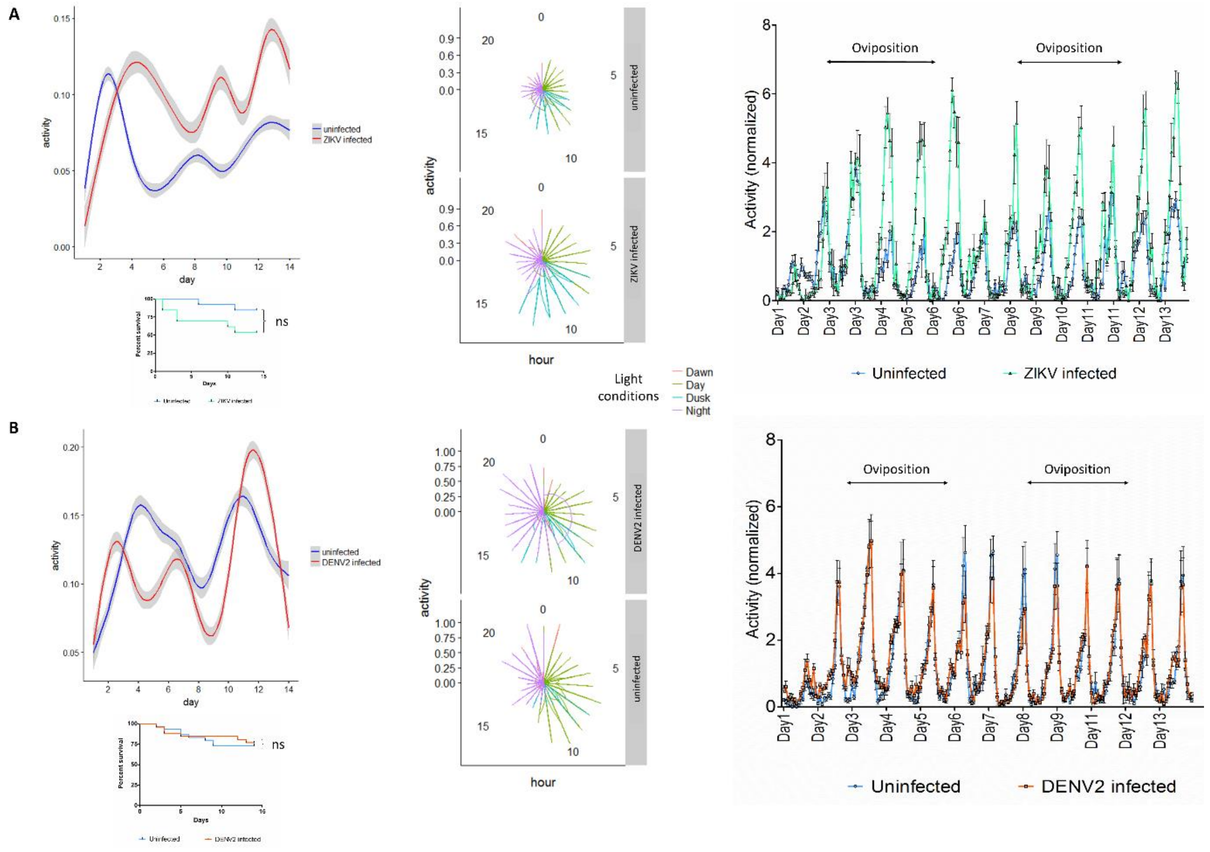


**Supplementary Figure S3. *Aedes aegypti* activity monitoring post infectious blood-feeding replicates**. Generalized additive modeling and for *Aedes aegypti* activity time series analysis *post* infectious blood-meal (0 dpi, first blood-meal) and non-infectious second blood meal (7 dpi) for ZIKV virus infection ($n$ = 13) (**A**) and DENV2 ($n$ = 25) (**B**). Each respective experimental duplicate has its corresponding polar activity in respect to light conditions, its survival curve (ns meaning "not significant") and its raw data line chart for mosquito female activity during the day (100% light) showing 24 hour oscillations. Each data point of the line chart represents one hour average of the sum of 10 minutes recording (±SEM). Two ZIKV and DENV2 infected groups are significantly different than uninfected with respectively $P < 0.0001$ (Two-tailed Wilcoxon signed rank test, W = -9625) and $P = 0.0003$ (Two-tailed Wilcoxon signed rank test, W = -5553).
