# Supplementary material for: Neurotropism and behavioral changes associated with Zika infection in the vector Aedes aegypti
Source: Emerg Microbes Infect. 2018 Apr 25;7:68. doi: 10.1038/s41426-018-0069-2 (PMC5915379; doi:10.1038/s41426-018-0069-2)
Supplement: Supplementary file 4 — Supplementary Figure S4 [file 41426_2018_69_MOESM4_ESM.pdf]

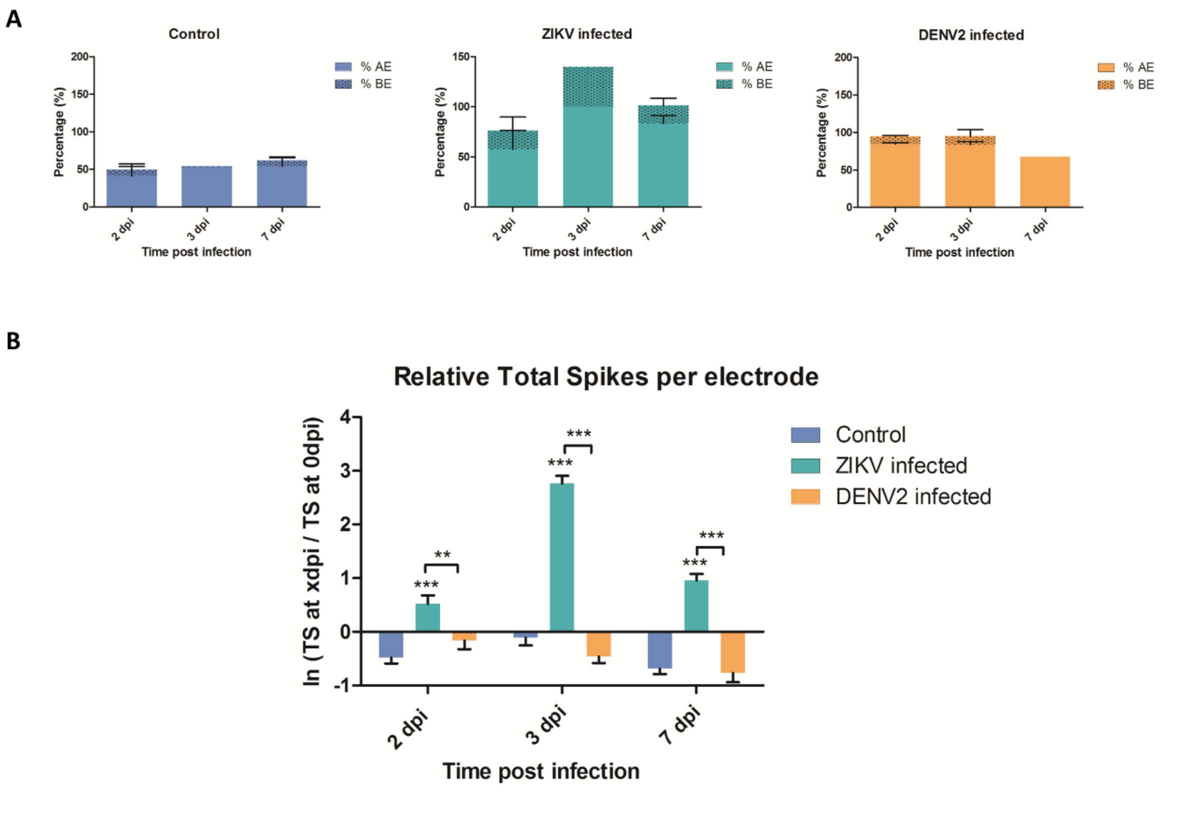

Supplementary Figure S4. Temporal analysis of *Aedes aegypti* neuronal network spontaneous activity on microelectrode array *post* infection. (**A**) Mosquito cultures bar plots representing the average proportion of Active Electrodes (AE) and Bursting electrodes (BE) per MEA for each group at different time points post ZIKV and DENV2 infections. (**B**) Bar plots representing the average ratio of total spike (TS) number per electrode between 0 dpi (reference) and TS number at 2, 3 and 7 dpi of spontaneous activity. All bar plots show mean value with ±SEM and statistical tests *p*-value are indicated on top of bars when compared to uninfected value. *T*-test results are summarized in Supplementary Table S4.
