# Supplementary material for: Neurotropism and behavioral changes associated with Zika infection in the vector Aedes aegypti
Source: Emerg Microbes Infect. 2018 Apr 25;7:68. doi: 10.1038/s41426-018-0069-2 (PMC5915379; doi:10.1038/s41426-018-0069-2)
Supplement: Supplementary file 5 — Supplementary Figure S5 [file 41426_2018_69_MOESM5_ESM.pdf]

A

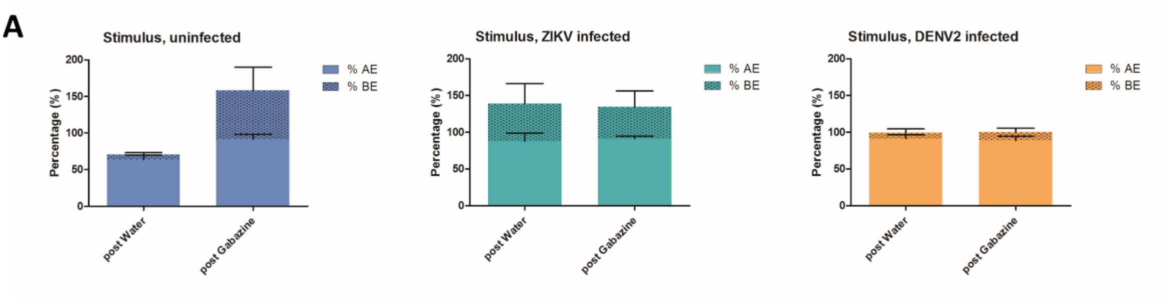

B

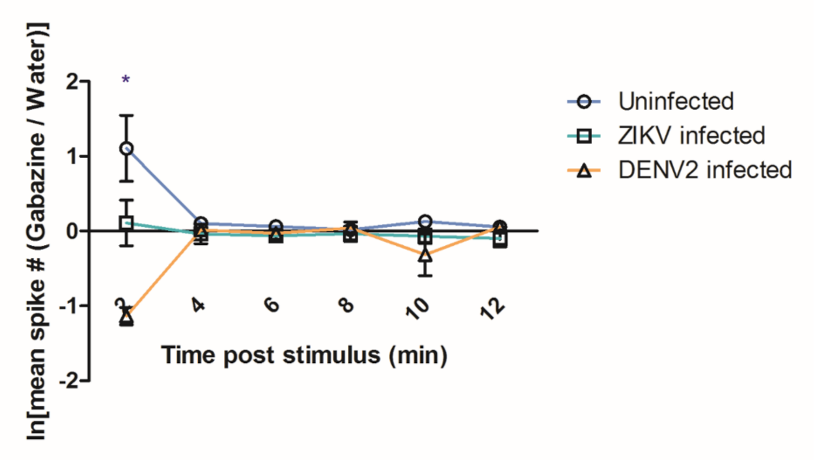

Supplementary Figure S5. GABA_A antagonist stimulus one week post infection on mosquito neuron cultures. (**A**) Bar plots representing the average proportion of Active Electrodes (AE) and Bursting electrodes (BE) per MEA for each group after introduction of the solvent (TC-water) and gabazine stimulus. (**B**) Line charts representing the average relative change of the mean spike number per electrode between the reference (TC-water) and the introduction of gabazine at different time points. All data points show mean value with ±SEM and statistical tests *p*-value are indicated on top of data points when significant.
