# Supplementary material for: Neurotropism and behavioral changes associated with Zika infection in the vector Aedes aegypti
Source: Emerg Microbes Infect. 2018 Apr 25;7:68. doi: 10.1038/s41426-018-0069-2 (PMC5915379; doi:10.1038/s41426-018-0069-2)
Supplement: Supplementary file 6 — Supplementary Figure S6 [file 41426_2018_69_MOESM6_ESM.pdf]

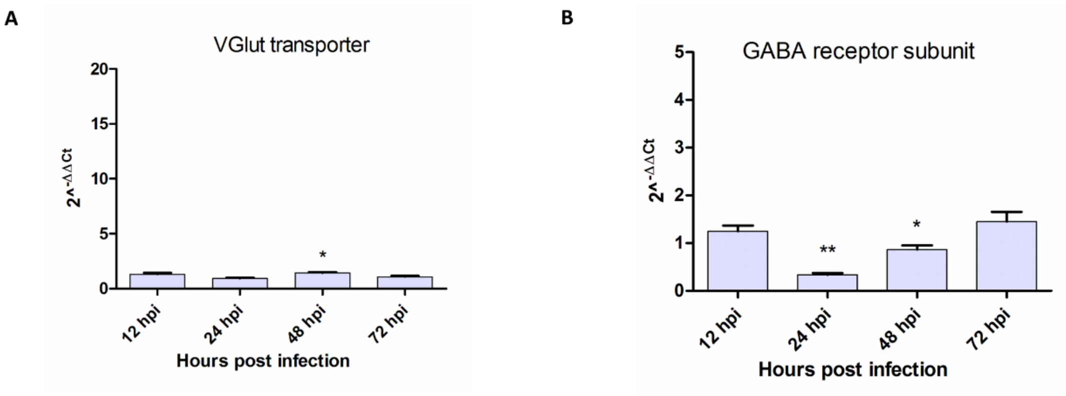

Supplementary Figure S6. Gene expression comparison after ZIKV infection in *Aedes aegypti* neuron cultures. Cultures were infected with ZIKV and real-time PCR was performed at various time points. (**A**) Bar plots illustrating mRNA expression of a Glutamate pathway protein: Glutamate transporter VGlut. (**B**) Bar plots illustrating mRNA expression of a $GABA_A$ pathway protein: $GABA_A$ receptor subunit. All bar plots show mean value with ±SEM (n = 3) of fold increase compared both to housekeeping gene and uninfected culture (ratio of $2^{-\Delta\Delta Ct}$) at the same time point in hours post infection (hpi). Statistical differences were calculated with unpaired t tests by comparing mRNA expression at $time_i$ to $time_{i-1}$ within each group.
