# Supplementary material for: Neurotropism and behavioral changes associated with Zika infection in the vector Aedes aegypti
Source: Emerg Microbes Infect. 2018 Apr 25;7:68. doi: 10.1038/s41426-018-0069-2 (PMC5915379; doi:10.1038/s41426-018-0069-2)
Supplement: Supplementary file 7 — Supplementary Figure S7 [file 41426_2018_69_MOESM7_ESM.pdf]

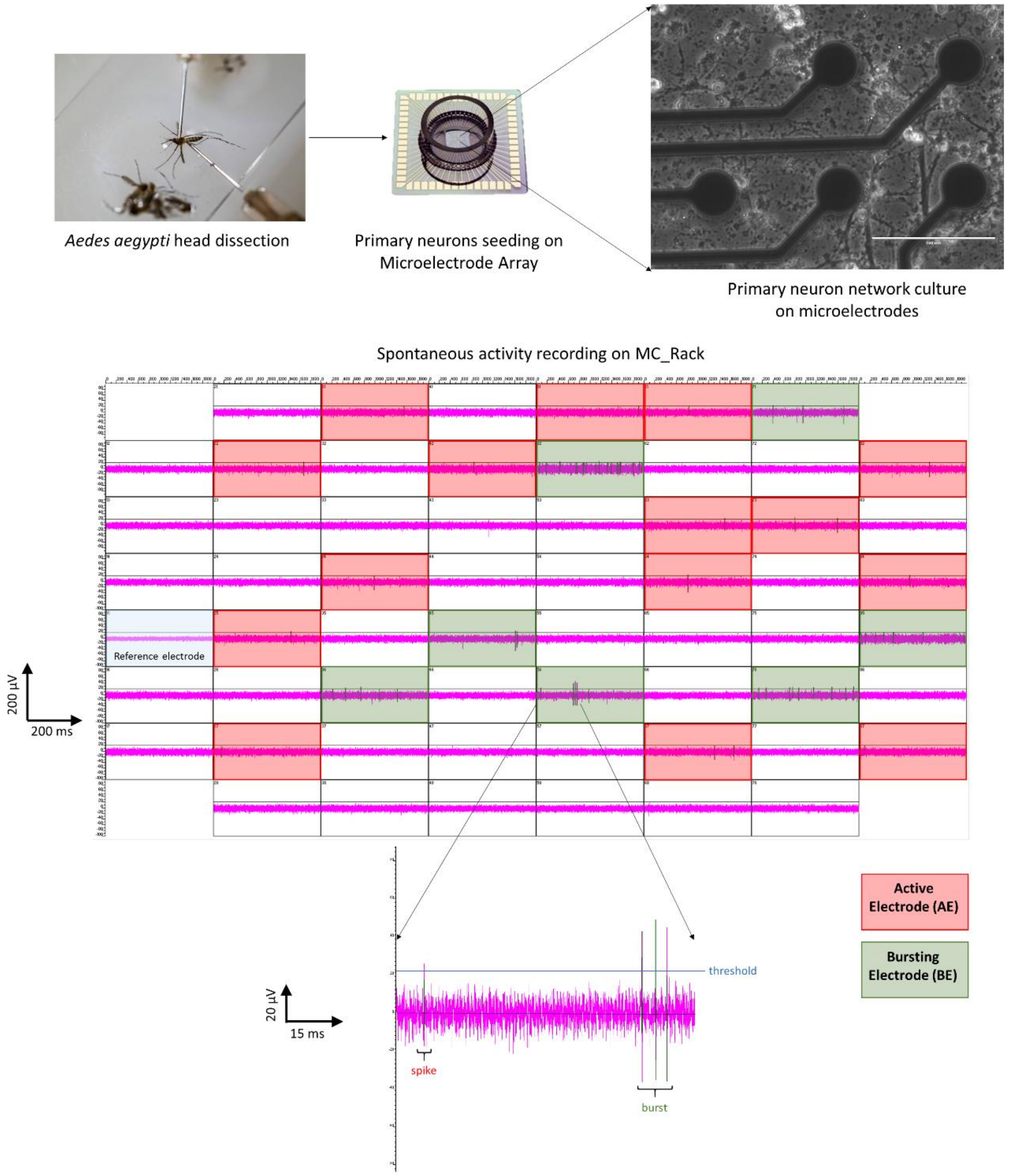

Supplementary Figure S7. Procedure of primary neuron culturing and recording on microelectrode array (MEA). Experimental setup and MEA analysis parameters illustration using *Aedes aegypti* primary neuron cultures on MEA.
