# Supplementary material for: Neurotropism and behavioral changes associated with Zika infection in the vector Aedes aegypti
Source: Emerg Microbes Infect. 2018 Apr 25;7:68. doi: 10.1038/s41426-018-0069-2 (PMC5915379; doi:10.1038/s41426-018-0069-2)
Supplement: Supplementary file 8 — Supplementary Table S1 [file 41426_2018_69_MOESM8_ESM.pdf]

Supplementary Table S1. Average number of eggs laid by female during each experimental assay.

		NUMBER OF EGGS PER FEMALE	
**EXPERIMENTAL ASSAY**	**Group**	**Blood-meal 1**	**Blood-meal 2**
**ZIKV_1**	**Uninfected**	19.5	37
	**Infected**	23.18	22.4
**ZIKV_2**	**Uninfected**	21.82	21.33
	**Infected**	17.56	23.25
**DENV2_1**	**Uninfected**	17.15	71.8
	**Infected**	23.08	70.86
**DENV2_2**	**Uninfected**	22.14	31.56
	**Infected**	10.62	19.29
