# Supplementary material for: Neurotropism and behavioral changes associated with Zika infection in the vector Aedes aegypti
Source: Emerg Microbes Infect. 2018 Apr 25;7:68. doi: 10.1038/s41426-018-0069-2 (PMC5915379; doi:10.1038/s41426-018-0069-2)
Supplement: Supplementary file 9 — Supplementary Table S2 [file 41426_2018_69_MOESM9_ESM.pdf]

Supplementary Table S2. Summary of statistical data analysis of mosquito neuron cultures spontaneous activity at different times post infection (Figure 2A)**.**

**Time line**	*2 dpi*	*3 dpi*	*7 dpi*
**Uninfected to ZIKV**	t = 5.097,<br>df = 407<br>$n_{uninfected}$ = 179<br>n_{ZIKV} = 230	t = 14.05,<br>df = 114<br>$n_{uninfected}$ = 57<br>n_{ZIKV} = 59	t = 10.61,<br>df = 446<br>$n_{uninfected}$ = 214<br>n_{ZIKV} = 234
	P < 0.0001	P < 0.0001	P < 0.0001
**Uninfected to DENV2**	t = 1.717,<br>df = 290<br>$n_{uninfected}$ = 179<br>n_{DENV2} = 113	t = 1.756,<br>df = 167<br>$n_{uninfected}$ = 57<br>n_{DENV2} = 112	t = 0.936,<br>df = 322<br>$n_{uninfected}$ = 214<br>n_{DENV2} = 110
	NS	NS	NS
**ZIKV to DENV2**	t = 2.828,<br>df = 341<br>n_{ZIKV} = 230<br>n_{DENV2} = 113	t = 16.27,<br>df = 169<br>n_{ZIKV} = 59<br>n_{DENV2} = 112	t = 8,<br>df = 342<br>n_{ZIKV} = 234<br>n_{DENV2} = 110
	P = 0.005	P < 0.0001	P < 0.0001
