# Supplementary material for: Neurotropism and behavioral changes associated with Zika infection in the vector Aedes aegypti
Source: Emerg Microbes Infect. 2018 Apr 25;7:68. doi: 10.1038/s41426-018-0069-2 (PMC5915379; doi:10.1038/s41426-018-0069-2)
Supplement: Supplementary file 10 — Supplementary Table S3 [file 41426_2018_69_MOESM10_ESM.pdf]

Supplementary Table S3. Forward (grey background) and reverse (white background) primers for mosquito (*Aedes aegypti*) genes used for Q-PCR.

***Aedes aegypti* (mosquito)**	
**Voltage-gated para Na channel (EU399179)**	CGGATATCGCGCTTCTCCAA
	ATGATTGTGCTGCTCACCTG
**Glutamate dehydrogenase 1 (XM_001660812)**	CGTTTCGTTCTTCGAGTGGC
	TTCCTGGATAGAGGCGAGCA
**EAAT (XM_001656303)**	TCGGAACCTCGTCAAGTTCG
	TCGGTAGCACAAATCGGGAC
**vGlut (XM_001654107)**	TTGAGGAAATCGAACGGCCA
	AATGATGAACCCCAGGCAGG
**GAT1 (XM_001660967)**	TCTTCATGGAACTGGCGCTT
	CAGCATGACATTACGGCTGC
**GABA receptor subunit XM_001653155 (U28803.1)**	ACGAATGCTGCCTTACCGAA
	GTGGCGTATTCCAGCAGACT
**RpS17**	AACGAAGCCCCTGCGCACAA
	CCTGCTCCAGGGCGGACACT
