# Supplementary material for: Neurotropism and behavioral changes associated with Zika infection in the vector Aedes aegypti
Source: Emerg Microbes Infect. 2018 Apr 25;7:68. doi: 10.1038/s41426-018-0069-2 (PMC5915379; doi:10.1038/s41426-018-0069-2)
Supplement: Supplementary file 11 — Supplementary Table S4 [file 41426_2018_69_MOESM11_ESM.pdf]

Supplementary Table S4. Summary of *n* used for statistical data analysis of confocal images.

		Fig. 3C	***Fig. 3D***
n, images	**0 dpi**	7	6
	**Uninfected**	13	12
	**ZIKV infected**	10	11
	**DENV2 infected**	12	11
n, total cells number	**0 dpi**	333	98
	**Uninfected**	565	240
	**ZIKV infected**	206	527
	**DENV2 infected**	565	397
n, coverslips	**0 dpi**	3	2
	**Uninfected**	4	4
	**ZIKV infected**	3	3
	**DENV2 infected**	3	3
